# Supplementary material for: Content-rich biological network constructed by mining PubMed abstracts
Source: BMC Bioinformatics. 2004 Oct 8;5:147. doi: 10.1186/1471-2105-5-147 (PMC528731; doi:10.1186/1471-2105-5-147)
Supplement: Additional File 2 — The original results of the above study (non-essential files are deleted to keep the file size under the limit set by BMC bioinformatics). [file 1471-2105-5-147-S2.bz2 › chilibotAdditionalFile2/dip10/273ID1922387E1093/html/MAPK1_JUN.html]

 


 **MAPK1** and **JUN** 
  
Found 2098 abstracts in PubMed, retrieved 10.  
 

 What does Google say? 
 PDF only 
| .edu only 

---

**Interactive relationship** (e.g. stimulation, inhibition, etc)

**Non-interactive relationship** (e.g. studied together, co-existance, homology, etc.)

- Sorbitol resulted in activation of the extracellularly responsive kinase ERK  [ **MAPK1** ] , p54 c  **Jun**  N terminal kinase JNK and protein kinase B PKB.  Ref: 12881532 J Biol ChemJ Biol Chem,
- We identified that serum starvation and Dox reduced phosphorylation of mitogen activated protein kinase extracellular signal regulated kinase ERK  [ **MAPK1** ]  kinase MEK, protein kinase C PKC alpha beta and c  **Jun**  NH 2 terminal kinase.  Ref: 12883037 Mol Cancer Ther, 2003
- Apoptosis signal regulating kinase1 ASK1 is a ubiquitously expressed mitogen activated protein kinase kinase kinase MAPKKK that activates the SEK1 c  **Jun**  N terminal kinase JNK and MKK3 MKK6 p38 MAPK  [ **MAPK1** ]  signaling cascades.  Ref: 12878192 Biochem Biophys Res Commun, 2003
- Such molecules include nuclear transcription factors e.g. GATA 4, c  **Jun** , protein kinases e.g. 90 kDa ribosomal protein S6 kinase, MAPK  [ **MAPK1** ]  activated protein kinase 2, and ion exchangers channels e.g. the Na H exchanger 1.  Ref: 12878473 J Mol Cell Cardiol, 2003
